# Cerebral Venous Sinus Thrombosis, a Nonenhanced CT Diagnosis?

**DOI:** 10.1155/2015/581437

**Published:** 2015-05-11

**Authors:** Ali Alsafi, Amish Lakhani, Lalani Carlton Jones, Kyriakos Lobotesis

**Affiliations:** Department of Neuroradiology, Charing Cross Hospital, Imperial College Healthcare NHS, London W6 8RF, UK

## Abstract

*Purpose*. Retrospectively evaluate the density of cerebral venous sinuses in nonenhanced head CTs (NCTs) and correlate these with the presence or absence of a cerebral venous sinus thrombus (CVST).* Materials and Methods.* Institutional review board approval was obtained and informed consent waived prior to commencing this retrospective study. Over a two-year period, all CT venograms (CTVs) performed at our institution were retrieved and the preceding/subsequent NCTs evaluated. Hounsfield Units (HUs) of thrombus when present as well as that of normal superior sagittal and sigmoid sinuses were measured. HU of thrombus was compared to that of normal vessels with and without standardisation to the average HU of the internal carotid arteries.* Results*. 299 CTVs were retrieved, 26 with a thrombus. Both raw and standardised HU measurements were significantly higher in CVST (*p* < 0.0001) compared to normal vessels. Both raw and standardised HUs are good predictors of CVST. A HU of ≥67 and a standardised measurement of ≥1.5 are associated with high probability of CVST on NCT.* Conclusion.* Cerebral venous sinus HU measurements may help improve sensitivity and specificity of NCT for venous sinus thrombosis and avoid potentially unnecessary follow-up examinations.

## 1. Introduction

Cerebral venous sinus thrombosis (CVST) is an uncommon cerebrovascular event, accounting for 0.5–1% of cases of stroke and affecting 1 in 500,000 people [1]. CVST is a disease of young adults (<50 years old) predominantly [2] and is diagnosed based on clinical suspicion with confirmatory neuroimaging [3]. Patients with CVST exhibit a wide range of nonspecific signs and symptoms creating a diagnostic challenge for the clinician and radiologist alike [3]. Headache is the commonest reported symptom in patients with CVST. It is present in ninety percent of cases and reflects raised intracranial pressure [2].

MRI is the noninvasive imaging technique of choice for diagnosing CVST, it is however not universally available in the acute setting [3–6]. CT venography (CTV) has now emerged as an alternative diagnostic test, which is at least as good as MRI and in some cases better, with the added advantage of being more readily available [7]. Previously, the gold standard for imaging and diagnosing CVST was digital subtraction cerebral angiography. This is not routinely used and has been superseded by CTV and MRV [3, 8–10].

As most patients with CVST present with nonspecific symptoms and often CVST is not immediately suspected, patients are likely to have nonenhanced head CT (NCT) at presentation. NCT is the examination of choice for screening patients with nonspecific neurological presentations in the context of low suspicion of CVST [4]. NCT may be reported as normal in up to two-thirds of patients with venous sinus thrombosis. When abnormal, the findings on NCT are often subtle and nonspecific in the early stages and include “hyper-dense” venous sinuses and cerebral swelling. Small recent studies have shown that venous sinus hyperdensity is a sensitive sign for CVST [11, 12]. Venous infarcts and fragmented haemorrhage are late signs [4].

Making a timely diagnosis of CVST is of utmost importance as prompt anticoagulation is thought to prevent thrombus propagation. This in turn prevents ensuing venous infarcts and haemorrhage thereby reducing mortality and long-term neurological sequelae [13]. There remains, however, much debate surrounding optimal management of patients with CVST.

## 2. Aim

In this study we sought to retrospectively evaluate the density (Hounsfield Units) of cerebral venous sinuses in nonenhanced head CT examinations in patients with and without a cerebral venous sinus thrombus as confirmed on CT venography. We also sought to evaluate whether standardising venous sinus HU measurements to those of the corresponding internal carotid arteries would improve diagnostic accuracy.

## 3. Materials and Methods

Institutional review board approval was obtained and informed consent waived prior to commencing this retrospective study.

All CTVs performed on adult patients in three teaching hospitals between September 2011 and November 2013 were retrospectively retrieved. These were reviewed and divided into positive CTVs and negative ones. The preceding or subsequent NCTs, within seven days of the CTV, were evaluated.

Only unique CTVs were included. Patients who underwent a neurosurgical procedure in the preceding 10 days were excluded as extra-axial blood products may affect our measurements. Follow-up CTVs, where the patient is known to have an old thrombus, were also excluded. We also excluded hypoplastic venous sinuses and heavily calcified ICAs from the analysis.

**Figure 1:**
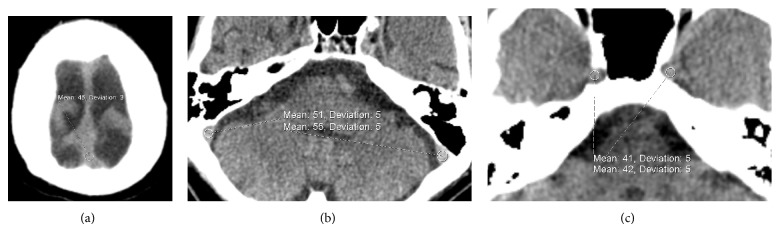
Images from NCT demonstrating sites of HU measurements from (a) superior sagittal sinus at the vertex, (b) sigmoid sinus, and (c) intracranial ICA just distal to the carotid canals.

The site of thrombus was identified from the CTV, where applicable, and the Hounsfield Units (HUs) of the corresponding cerebral venous sinus in the NCT were measured. In negative studies, HU of the superior sagittal sinus (SSS) and both sigmoid sinuses were recorded (Figure 1).

In both groups, HUs of both intracranial portions of the internal carotid arteries (ICAs) as they exit the carotid canal were measured (Figure 1).

The HUs of the cerebral venous sinuses with and without thrombus were compared before and after normalisation to the average HU of the corresponding ICAs (see the following equation):

(1) Standardised measurment=HU of venous sinusHU of right ICA+HU of left ICA/2.

In our institution patients with CVST are anticoagulated with unfractionated heparin acutely in the absence of contraindications then changed to warfarin once stable and ready for discharge.

GraphPad Prism 5.0 for Mac OS X (GraphPad Software Inc., San Diego, CA, USA) was used for statistical analysis. Student's* t*-test was used to compare HUs. Fischer's exact test was used to compare categorical data.

Receiver operating characteristic (ROC) curves were derived and the area under the ROC curves (AUC) was calculated. 95% confidence intervals were used to test the hypothesis that the AUC is 0.5.

Microsoft Excel for Mac 2011 v 14.0.0 (Microsoft Corporation, One Microsoft Way, Redmond, WA) was used to compare the AUCs of unpaired ROC curves (raw HU versus per patient HU and standardized HU versus per patient HU) using the method described by Hanley and McNeil [14].

MedCalc version 12.7.0 (MedCalc Software, Acacialaan 22, 8400 Ostend, Belgium), was used to compare the AUC of paired ROC curves, that is, raw versus standardized HU [15].* p* values < 0.05 were considered significant.

### 3.1. NCT and CTV Protocols

#### 3.1.1. CTV Protocol

Axial NCTs were performed on Somatom Definition AS CT scanners (Siemens Medical Solutions, Forchheim, Germany) with the following parameters: 320–360 mAs, 120 kV, and slice collimation of 12 × 0.75 mm for the posterior fossa and 12 × 1.5 mm for the rest of the brain.

CTVs were performed 45 s after administration of intravenous iodinated contrast at 3/4 mL/s. The detector configuration was 16 × 0.75 mm.

All scans were performed from the top of the C1 lamina to the top of the calvarium, parallel to the floor of the anterior fossa avoiding the eyes.

## 4. Results

299 CTVs were retrieved. 22 postoperative (postneurosurgical procedure) studies were excluded. 24 studies were excluded, as there was no preceding or follow-up NCT within seven days of the index CTV. Five hypoplastic venous sinuses and six heavily calcified ICAs were excluded from analysis.

The remaining 250 unique patients with a median age of 42 had a CTV and NCT within seven days of each other with a median of 0 days and a range of [−1, 6.6]. 148 patients had a CTV immediately following their NCT. Two patients had an NCT 24 hours following CTV. No patients underwent an NCT less than 24 hours following their CTV.

26 patients had a CTV proven CVST with a median age of 38.7 while 224 patients had normal CTVs; they had a median age of 43.3. 19% (5/26) of those with a CVST an acute intracranial haemorrhage evidence on NCT, compared with 15% (33/224) from the normal CTV group. Only 10.4% of patients with a suspected CVST had a proven thrombus on CT venography. There was no significant difference in age, sex, or incidence of intracranial haemorrhage between the two groups with *p* values of 0.3800 (two-tailed Student's* t*-test), 0.2953, and 0.5641 (Fisher's exact test), respectively. Demographic data are shown in Table 1 and distribution of CVST is shown in Table 2.

### 4.1. Vessel HU Analysis

**Figure 2:**
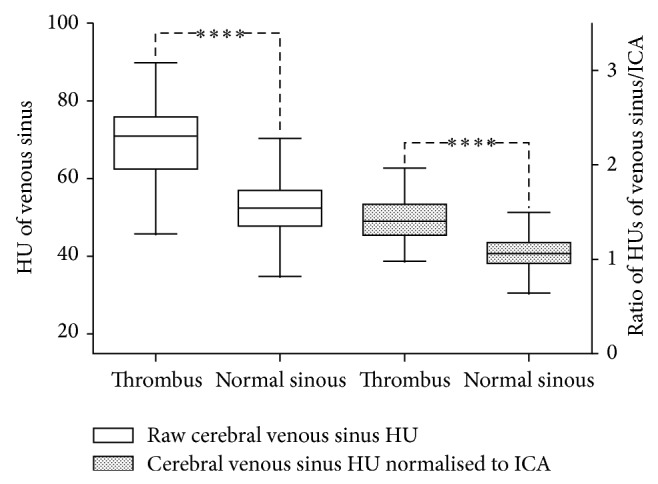
Comparison of cerebral venous sinus HU before and after standardisation to the ICAs in patients with and without CVST. ∗∗∗∗ = *p* < 0.0001 two-tailed Student's* t*-test.

768 venous sinuses were analysed, 46 with a CVST and 720 without. The average HU of vessels containing a thrombus was 68 ± 1.56 (*n* = 46) which was significantly higher than that of normal vessels 52 ± 0.28 (*n* = 720) (*p* < 0.0001, two-tailed* t*-test) (Figure 2).

In an attempt to eliminate HU fluctuations caused by low haemoglobin, haemodilution, dehydration, and so forth, we standardised the venous sinus HUs to the average HU of the corresponding ICAs.

The ratio of sinuses containing thrombus was 1.44 ± 0.04 (*n* = 46). The ratio in sinuses without thrombus was 1.07 ± 0.01 (*n* = 720). This was significantly different (*p* < 0.0001 by the two-tailed* t*-test).

### 4.2. Diagnostic Performance of Raw versus Standardized Measurements

**Figure 3:**
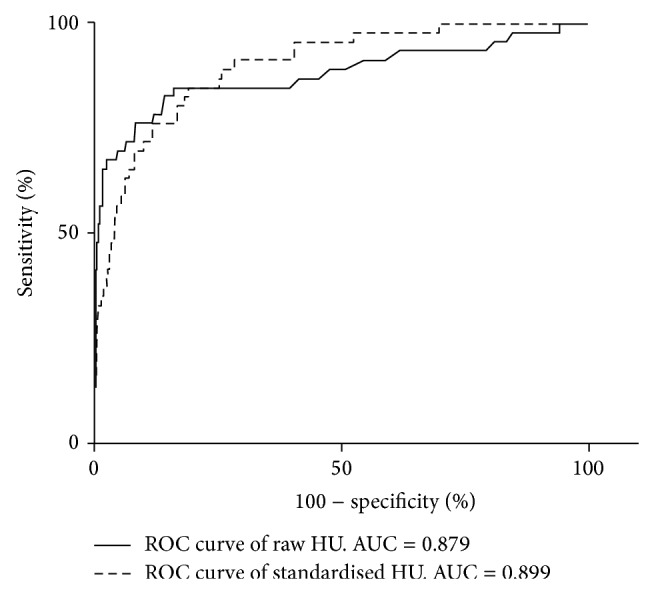
ROC curves comparing the difference between raw HU and standardized measurements.

ROC curves were derived from the raw and standardized HUs (Figure 3). The diagnostic performance of raw venous sinus HUs and standardized HUs expressed as the area under the curve (AUC) were 0.899 (95% CI [0.875–0.919]) and 0.879 (95% CI [0.853–0.901]), respectively. The AUCs show that both measurements were good predictors of CVST (*p* < 0.0001). Standardized HU measurements perform slightly better compared with raw HU albeit not reaching statistical significance (*p* = 0.6078).

## 5. Discussion

Although nonenhanced head CT is thought to have a low sensitivity and specificity for CVST, Roland et al. estimate the sensitivity at 73% [16]. This may be even lower in normal clinical practice, when CVST is not suspected nor mentioned in the clinical detail.

In a recent small study, cerebral venous sinus measurements in NCT were shown to be of value in detecting CVST [12]. The study included a small number of controls without venous sinus thrombus and their suggested cutoff HU of 62 is likely to yield a significant number of false positive studies resulting in unnecessary follow-up examinations [12]. Our study has a larger control population showing wide variation in normal venous sinus HU and is likely to be more reflective of general clinical practice.

Black et al. have previously demonstrated a significant difference in venous sinus densities between patients with and without CVST, although they assumed that patients with a normal NCT have no CVST without a confirmatory CTV or MRV [11]. Our results more robustly confirm these findings as all our patients have had a CTV. Our results show that when assessing cerebral venous sinuses on NCT, HU measurements are a useful objective adjunct to subjective assessment in detecting CVST.

Black et al. have also demonstrated a correlation between the patient's hematocrit and venous sinus density [11]. With that in mind, we standardised venous sinus HU measurements to those of the average ICAs. We postulated that standardization may eliminate some of the physiological variations encountered such as haemodilution, dehydration, and anemia.

The area under the ROC curves for the standardized HUs was 0.899 (95% CI [0.875–0.919]) compared with 0.879 (95% CI [0.853–0.901]) for raw HU measurements although the difference did not reach statistical significance (*p* = 0.6078) using DeLong's paired measurements ROC analysis [15]. Both methods have a strong correlation with the presence or absence of CVST with *p* < 0.0001.

Black et al. also noted that patients with a CVST often had a HU > 70, whereas most patients without a CVST had HU < 70 and also showed that a HU/hematocrit ratio of >2 has a good correlation with CVST. Our results, on the other hand, demonstrate that using 70 as a cutoff is likely to result in a significant number of false-negative studies.

A recent study demonstrated that the sensitivity of venous sinus hyperdensity on NCT may be as high as 100% with a specificity reaching 95%. This is also likely to be an overestimate as the reading radiologists had only one task of visually scoring the venous sinuses on NCTs [17].

**Figure 4:**
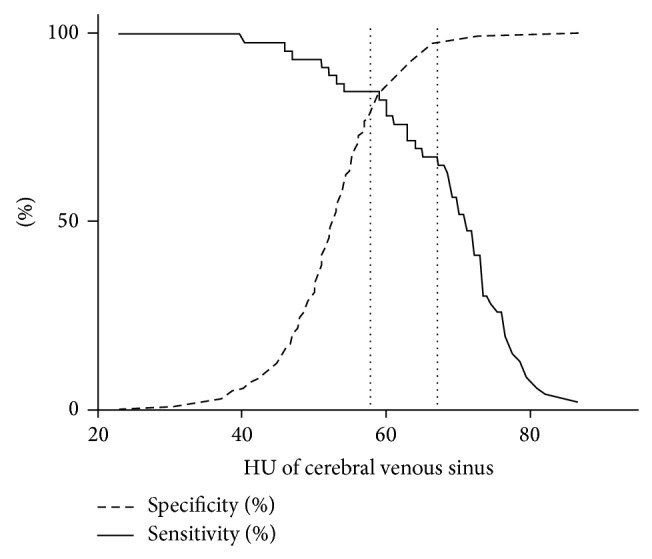
Graph demonstrating changes in sensitivity and specificity at different HU cutoffs for standardised HU measurement. The dotted lines represent our proposed HU cutoffs with high negative and positive predictive values for venous sinus thrombosis.

Cerebral venous sinuses with a HU of <58 are associated with a low probability of CVST (likelihood ration (LR) of 0.187). A venous sinus with HU of ≥67 on the other hand is associated with a high probability of CVST (LR 27.621). A cerebral venous sinus with a CVS/ICA ratio of <1 is associated with a low probability of CVST (LR 0.0619), while a CVS/ICA ratio of ≥1.5 yields a LR of 25.043 and a high probability of CVST (Figure 4).

Old thrombus however may also have a low HU and may be missed despite HU measurements. If the patient's symptoms are long standing, a CTV or MRV may still be warranted. CVST in anaemic patients and those with low haematocrit may also be of low density. Low HU measurements cannot exclude a thrombus in such cases.

The aim of this study is not to provide an alternative to CT or MR venography but to provide the reader with more confidence in their observations on NCT. It remains imperative for the reader to analyse the NCT images as usual, but with the added benefit of being able to objectively assess the density of a suspicious venous sinus. Although venous sinus HU measurements may be helpful when interpreting a CT, they rely on the reader's judgment initially as one will not routinely sample every cerebral venous sinus to assess HU. Instead, only when there is concern regarding a venous sinus will the reader proceed to HU measurements.

The number of positive cases in this study is relatively small potentially affecting the generalizability of our data. This is a function of CVST being a rare diagnosis. A larger study with multicentre collaboration may be more useful to assess the generalizability of our data.

The fact that we did not use the raw CT data may introduce some errors into our HU measurements. We decided to use this for practical reasons, as the raw data was not readily available, which is the case in usual clinical practice. Despite this HU we showed a good correlation between HU measurement with and without standardisation and CTV results making it a clinically useful, practical tool. Although, HU measurements correlate well with CTV findings; one cannot extrapolate from our results that this can translate into better pick-up rates of CVST from NCT. To assess this, a further study is needed to assess if using HU measurements improves the radiologist's pick-up rate of CVST.

Objective HU measurements of suspicious cerebral venous sinus appearances on NCT may provide an additional tool in the radiologist's arsenal to increase reporting confidence of the relatively rare clinical entity of cerebral venous sins thrombosis guiding further imaging and management.

## Figures and Tables

**Table 1:** Patients' characteristics.

	CVST (*n* = 26)	No CVST (*n* = 224)
Median age	38.7 [21.1–63.6]	43.3 [17.3–88.7]
Sex	M = 13, F = 13	M = 87, F = 137
Haemorrhage	5	33
Same day NCT and CTV	21	127

**Table 2:** Distribution of CVST.

Vessel	Number of vessels with thrombus
Internal jugular vein	4
Transverse sinus	15
Sigmoid sinus	14
Superior sagittal sinus	11
Torcula herophili	2
Total vessels with CVST	**46**

CT: computed tomogaphy, NCT: non-enhanced CT, CTV: CT venogram, CVST: cerebral venous sinus thrombosis.

## Abbreviations

CT: Computed tomography
NCT: Nonenhanced CT
HU: Hounsfield units
ICA: Internal carotid artery
CVST: Cerebral venous sinus thrombus
AUC: Area under the curve
ROC: Receiver operating characteristic.
